# The RNA-Binding Motif Protein Family in Cancer: Friend or Foe?

**DOI:** 10.3389/fonc.2021.757135

**Published:** 2021-11-04

**Authors:** Zhigang Li, Qingyu Guo, Jiaxin Zhang, Zitong Fu, Yifei Wang, Tianzhen Wang, Jing Tang

**Affiliations:** ^1^ Department of Orthopedics, Affiliated Hospital of Chifeng University, Chifeng, China; ^2^ Department of Pathology, Harbin Medical University, Harbin, China; ^3^ Department of Urology, Hainan General Hospital, Hainan, China

**Keywords:** RNA-binding motif protein, cancer, prognosis, tumorigenesis, RNA binding protein

## Abstract

The RNA-binding motif (RBM) proteins are a class of RNA-binding proteins named, containing RNA-recognition motifs (RRMs), RNA-binding domains, and ribonucleoprotein motifs. RBM proteins are involved in RNA metabolism, including splicing, transport, translation, and stability. Many studies have found that aberrant expression and dysregulated function of RBM proteins family members are closely related to the occurrence and development of cancers. This review summarizes the role of RBM proteins family genes in cancers, including their roles in cancer occurrence and cell proliferation, migration, and apoptosis. It is essential to understand the mechanisms of these proteins in tumorigenesis and development, and to identify new therapeutic targets and prognostic markers.

## Introduction

RNA-binding proteins (RBPs) are a kind of crucial intracellular protein, which can be widely involved in a variety of post-transcriptional regulation processes, such as RNA splicing, transport, localization, and translation. RBPs are divided into many kinds according to different functions, including Hu-antigen R (HuR), heterogeneous nuclear ribonucleoprotein family (hnRNP), the arginine/serine-rich splicing factor protein family (SRSF), and RNA-binding motif (RBM) proteins family, etc. (1). RBM proteins family is a subgroup of RBPs, which has the same domain characteristics as RBPs, including RNA-recognition motifs (RRMs), RNA-binding domains (RBM), ribonucleoprotein (RNP), cold-shock domain (CSD), and zinc finger (ZnF), etc. (1). RRM is a central structural motif of the RBM proteins family; usually, RBM protein has one or more RRMs, such as RBM3, which includes one RRM, and RBM19 contains up to six RRMs. The member of the RBM proteins family is named sequentially after confirming that they contain RRM. Up to now, more than 50 RBM proteins have been identified (**Table S1**). It is worth noting that not all the RRM-containing RNA-binding proteins are designated as RBM proteins. Once the exact functions of the RBM protein are determined, the RBM protein will be renamed according to its function, and the ‘‘RBM’’ designation can be removed (2).

**Figure 1:**
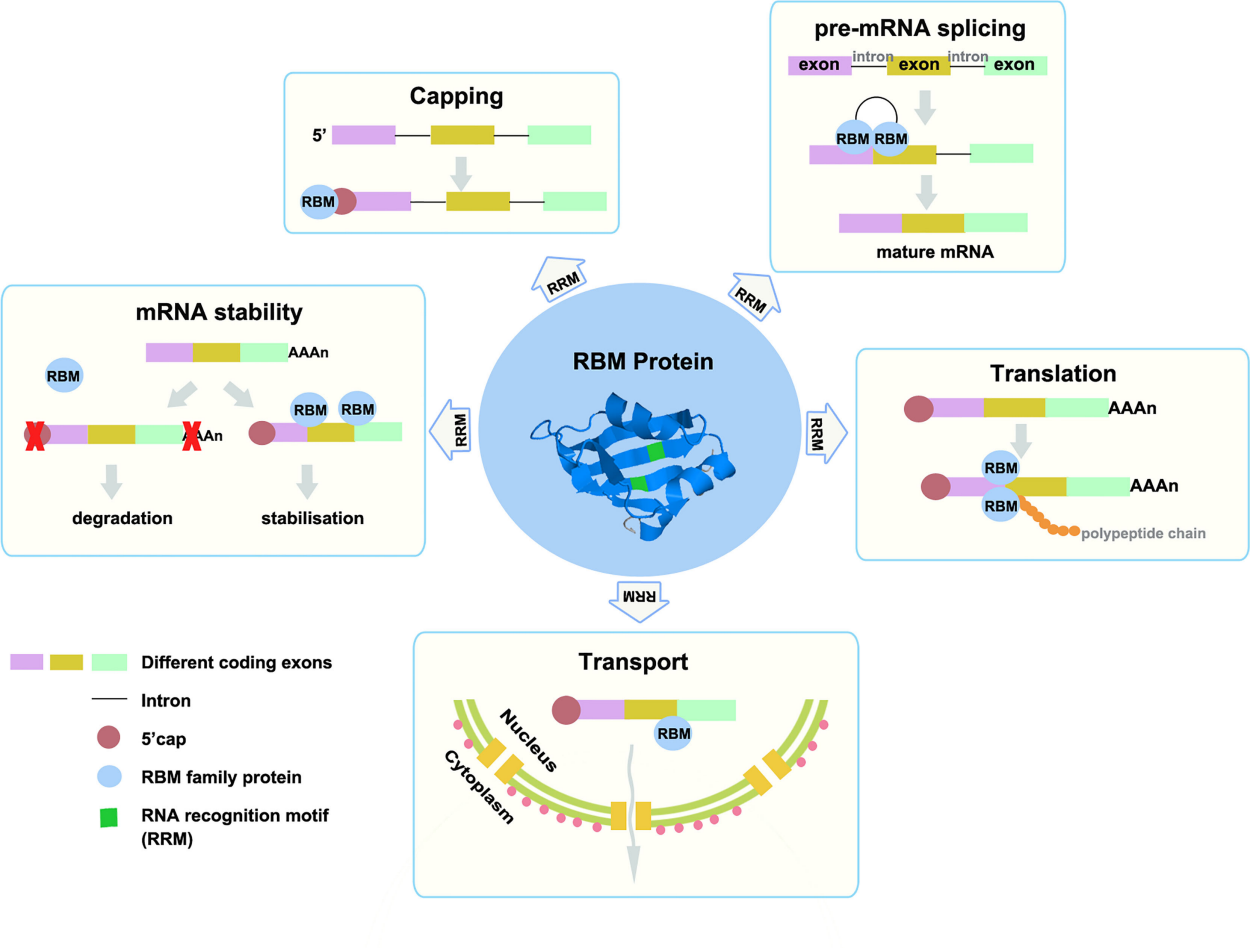
The RBM proteins family can affect gene expression and function by intervening mRNA transport, translation, capping, splicing, and stability. The three colored rectangles represent different exons, black lines represent introns, reddish brown circles represent 5 ‘cap, light blue circles represent RBM family proteins, and green rectangles represent the RRM domain. The central circle is the structure of RBM protein, usually, it has one or more RRMs.

Like RBPs, the RBM proteins family are involved in multiple biological activities, such as RNA metabolism, including pre-mRNA splicing, RNA stability, mRNA translation, etc. (**Figure 1**) (3–7). RBM proteins can regulate alternative splicing by binding to the exon/intron region near the splice site of mRNA. For example, RBM10 can bind to the intron region near the splice site on mRNA, thus interfering with the recognition of splice site, while RBM5 and RBM6 can bind to the exon region near the splice site and recruit splicing components (8). And RBM4 has been reported can regulate the selection of 5’ splice sites or exons *in vitro* and antagonize the effect of SRSF protein on the selection of 5’ splice sites (9). In addition, RBM proteins can regulate the stability of RNA by directly binding its target mRNAs, such as RBMS1, RBM38, and RBM3 (10–13). Among them, RBM3 also participates in the translation regulation of cyclooxygenase-2 (COX-2) mRNA by recognizing and binding COX-2 AU-rich elements (ARE) sequence. Overexpression of RBM3 can improve the mRNA translation of COX-2 in HCT116 cells (13). Over the past few decades, different effects of the RBM proteins family have been gradually found in various cancer-related studies. In this review, we focused on the role of the RBM proteins family in cancer and summarized the effects of the RBM proteins family members on the occurrence, progression, and treatment of cancer.

## RBM Proteins Family Is Frequently Related to the Occurrence of Cancer

Several studies showed that the RBM proteins family is closely related to the occurrence of cancer. RBM3, a cold-induced RNA-binding protein, was found to be upregulated in several types of cancers (14–17). However, Zeng et al. found that the overexpression of RBM3 in PC3 cells (a human prostate cancer cell line) weakened the stem cell-like characteristics of these cells (18). They indicated that RBM3 hindered the occurrence of prostate cancer because the tumor formation rate of PC3 cells overexpressed with RBM3 in nude mice was significantly lower than that in the control group (18). p53 is the most common mutant gene in human cancer and mutant p53 has been reported to promote tumor metastasis. RBM38, also known as RNPC1, is a target gene of the p53 family; it can inhibit p53 translation by interacting with eIF4E on p53 mRNA (11, 19, 20). Zhang et al. found that RBM38 can jointly regulate mutant p53 and PTEN, a key regulator of T cell development, to affect the occurrence of T cell lymphomas. They showed that the deletion of RBM38 enhanced the expression of mutant p53, and decreased the expression of tumor suppressor PTEN, which promoted the occurrence of lymphoma (21). And Zhang et al. found that mice who deleted RBM38 were more prone to aging and spontaneous tumors (20). These researches indicated that RBM38 could interact with p53 to form a negative feedback regulatory loop to involve tumorgenesis. RNA-binding motif single-stranded interacting protein 3 (RBMS3), another member of the RBM proteins family, has been reported could suppress the morphogenesis of non-small cell lung cancer (NSCLC) (22). Based on these studies, some RBM proteins, such as RBM38, RBM3, and RBMS3, play an inhibitory role in tumorigenesis. However, whether other RBM proteins family have the same effect in tumorigenesis has not been reported. Therefore, the role of the RBM proteins family in tumorigenesis and related molecular mechanisms still needs to be further explored.

### RBM Proteins Family Can Promote Tumor Cell Proliferation

Studies have found that RBM proteins family can promote the proliferation of tumor cells. The mechanism of the RBM proteins family promoting proliferation is complex and usually involves the following aspects.

#### RBM Proteins Family Can Affect Tumor Cell Proliferation by Regulating Cancer-Related Genes and Signaling Pathways

SM Sureban et al. reported that RBM3 could promote the proliferation of colon cancer cells by enhancing the stability and translation ability of COX-2, IL-8 and VEGF mRNA (13). Hypoxic and other adverse conditions that are detrimental to cell growth, RBM3 participates in the survival of colon cancer cells mainly through a COX-2 signal transduction mechanism (23). Furthermore, RBM3 could promote the growth and proliferation of hepatocellular carcinoma (HCC) cells in the stearoyl-CoA desaturase (SCD)-circRNA-2-dependent manner by control SCD-circRNA-2 formation (24). Lin et al. found that RBM4 inhibits the apoptosis of breast cancer cells by upregulating the expression of transcripts IR-B and MCL-1S (25). In the U251 cell line, RBM17 decreased the expression of apoptosis related factors caspase 3, caspase 9 and PARP, and promoted the proliferation of glioma cells (26).

#### RBM Proteins Family Can Promote Cell Proliferation by Participating in the Regulation of Cell Cycle

HAN et al. found that inhibiting RBM17 expression can significantly reduce the proliferation of hypopharyngeal carcinoma cells, promote their apoptosis, and block their cell cycle progression at the G2/M phase (27). RBM17 plays a similar role in HCC and glioma. Li et al. showed that inhibiting RBM17 expression can decrease the proliferation of HCC cells, arrest cells at the G2/M phase, and significantly increase the apoptosis rate (28). In breast cancer cells, knocking down the RBM7 gene also inhibits cell proliferation, and induces G1 cell cycle arrest. Whereas overexpressing RBM7 promotes the proliferation of breast cancer cells by binding to AU-rich elements of cyclin-dependent kinase1 (CDK1) 3’-UTR and then stabilizing CDK1 mRNA (29).

Other RBM proteins family members also participate in promoting proliferation in various cancers, such as RBM5-AS1, RBM11, RBM15, RBM23, RBM33, etc. (**Table 1**). The proliferative effects of the RBM protein family members, as mentioned above that on tumor cells, may contribute to tumor progression. Nevertheless, the RBM proteins family can also play anti-tumor effects in cancers.

**Table 1:** The RBM family proteins effect promotes a role in cancers.

RBM family protein	Cancers	The role of RBMs in the cancer	Relative molecular mechanism	Associate cell lines or animal models	PMID
RBM5-AS1	Hepatocellular carcinoma	RBM5-AS1 knockdown dramatically restrains cell proliferation, invasion and migration of HCC cells.	RBM5-AS1 acts as an epigenetic regulator to promote the HCC progression by repressing miR-132/212 expressions.	Normal human hepatocytes (LO2) and HCC cell lines(Huh7, HepG2, Hep3B, Bel-7405 and SMMC-7721)	34019714
	Osteosarcoma	RBM5-AS1 promoted Os cell proliferation, migration, and invasion.	RBM5-AS1 targeted RBM5, but the underlying mechanism is still unclear, and needs further research.	Os cell lines (MG63, U2OS, SAOS2, HOS, 143B)and the normal osteoblast cell line (hFOB1.19)	33816613
	Oral squamous cell carcinoma	RBM5-AS1 promotes the proliferation, migration, and invasion of OSCC cells.	RBM5-AS1 regulates the level of miR-1285-3p as a competitive endogenous RNA (ceRNA), therefore regulate the expression level of an oncogene-YAP1, a target of miR-1285-3p.	OSCC cancer cell lines (Tca8113, SCC9, SCC25, CAL27, HN12, HSU3, FADU) and normal human oral kerati nocytes cell (NHOK ).	31869662
RBM7	Breast cancer	RBM7 promotes breast cancer cell proliferation.	RBM7 promoted breast cancer cell proliferation by stabilizing CDK1 mRNA via binding to AREs in its 3'-UTR.	Breast cancer cell lines (SUM-1315, MCF-7, BT474, ZR-75-1, and MDA-MB-231)	33145401
RBM11	Ovarian cancer	RBM11 promotes ovarian cancer cell growth and invasion.	RBM11 promotes ovarian cancer progression through stimulating Akt/mTOR signaling pathways.	Ovarian cancer cells (A2780 and OVCAR-3)	34434291
RBM15	Chronic myelogenous leukemia	Knockdown of RBM15 slows cell growth and induces apoptosis in chronic myelogenous leukemia cells.	Knockdown of RBM15 could induce G1 → S phase arrest in chronic myelogenous leukemia cells.	Erythroleukemia cell line (K562)	22497198
	Laryngeal squamous cell carcinoma	RBM15 promotes LSCC cells migration and invasion.	TMBIM6 acted as a downstream target of RBM15-mediated m6A modification. Furthermore, RBM15-mediated m6A modification of TMBIM6 mRNA enhanced TMBIM6 stability through IGF2BP3-dependent.	LSCC cells (AMC-HN-8 cells, TU-212 cells, and TU-177 cells) and normal human bronchial epithelial cell (NHBEC)	33637103
RBM17	Glioma	RBM17 functions in promoting cell proliferation, affecting the cell cycle, and inducing apoptosis in human glioma cells.	RBM17 decreased the expression of Caspase-3, Caspase-9, and PARP and active cleaved caspase-3 and cleaved PARP in the U251 cell line. RBM17 was capable of regulating these apoptosis-related factors	Glioma cell lines (U251 and U87)	30227940
	Hepatocellular carcinoma	RBM17 silencing can inhibit cell proliferation.	RBM17 knockdown arrested the progression of the cell cycle, causing cells to halt at the G2/M phase.	HCC cell lines (Hep3B, SKHEP-1, Huh7, HepG2, HCC-LM3, SMCC-7721, BEL-7402, and MHCC-97L. SKHEP-1)	32497093
	Hypopharyngeal carcinoma	Knockdown of RBM17 inhibits growth of hypopharyngeal carcinoma cells.	The knockdown of RBM17 increased the proportion of cells undergoing apoptosis and arrested the cell cycle at the G2/M phase.	Hypopharyngeal carcinoma cell lines (FaDu)	29552202
RBM23	Hepatocellular Carcinoma	Knockdown RBM23 expression of HCC cells significantly inhibited the tube formation by the human vascular endothelial cells in vitro.	RBM23 activated the NF-κB signaling pathway and promoted expression of the proangiogenic cytokines selectively.	HCC cell lines (Huh-7, SK-HEP-1, SMMC-7721, and HepG2)	33791378
RBM33	Gastric cancer	circRBM33 promotes tumor cell proliferation, migration, and invasion.	circRBM33 facilitates the progression of GC through binding with miR-149 and modulating IL-6 levels.	GC cell lines (AGS, SGC-7901, BGC-823, MGC-803) and healthy gastric epithelial cells (GES-1)	32044717
	Cervical cancer	circRBM33 exerted a promoting influence on the malignant behaviors and glycolysis of cervical cancer cells.	CircRBM33 fostered CC advancement via absorbing miR-758-3p and upregulating PUM2.	CC cell lines (HeLa and SiHa) and normal cervical epithelial cells (ECt1/E6E7)	33398465
RBM39	Breast cancer	RBM39 depletion reduces tumorigenesis and cancer hallmarks of breast cancer cells.	RBM39 functions as a master transcriptional regulator that interacts with the MLL1 complex to facilitate chromatin binding and H3K4 trimethylation in breast cancer cells.	Breast cancer cell lines (T47D, HCC1428, ZR7530, HCC1954, HCC2157, DU4475, HCC1395, HCC38,MDA-MB361, MDA-MB453, MDA-MB468, MDA-MB231 and Jurkat)	34077726

### RBM Proteins Family Inhibits Tumor Cell Proliferation

To date, studies on the anti-tumor proliferation effects of the RBM proteins family on cancer have mainly focused on the following aspect.

#### RBM Protein Inhibits Cell Proliferation by Targeting the Expression of Proto-Oncogene or Anti-Oncogene

RBM38 can suppress c-Myc protein expression to suppressed cell proliferation by directly binding to target AU-rich elements in the 3’-UTR of c-Myc mRNA. Conversely, c-Myc negatively regulates RBM38 expression by binding to the E-box in the promoter region of the RBM38 gene in breast cancer (30). RBM38 can also increase the expression of phosphatase and tensin homolog gene on chromosome 10 (PTEN) by binding to the 3’-UTR of PTEN transcript, thereby inhibiting the cell proliferation of breast cancer (31). Zhang et al. reported that RBM38 is phosphorylated at Ser195 by glycogen synthase kinase 3 (GSK3), promoting the translation of p53 mRNA and inhibiting tumor cell growth and proliferation (32).

#### RBM Proteins Family Can Inhibit Cell Proliferation by Regulating the Cell Cycle in Cancer

P21 protein is a cyclin-dependent kinase inhibitor that can arrest the cell cycle and prevent cell proliferation. RBMS2 positively regulates the stability of P21 mRNA by binding to its 3’ -UTR and therefore inhibits the proliferation of breast cancer cells (33). RBM43 is another tumor suppressor gene in the RBM proteins family. It is significantly downregulated in tumors, and its low expression is associated with a poor prognosis (34). Overexpression of RBM43 can inhibit the cell cycle progression by directly binding to the 3’ -UTR of CyclinB1 and then reducing CyclinB1 expression in HCC cells (34).

#### RBM Protein Can Inhibit Tumor Proliferation by Regulating Signal Pathway

Yong et al. found that RBM4 can inhibit the proliferation of gastric cancer cells *in vitro* and *in vivo*. RBM4 inhibits the activity of MAPK dependent signal pathway by inhibiting the expression of MAPK pathway protein, so it plays a role in inhibiting the proliferation of gastric cancer cells (35). RBM5 is a tumor suppressor gene in lung cancer and breast cancer, but its role in the pathogenesis of medulloblastoma (MB) remains unclear. Yu et al. Found that RBM5 knockdown induced Daoy and ons-76 cells proliferation, and the β-Catenin protein expression level was up-regulated in Daoy cells, Therefore, RBM5 may regulate Wnt/β- Catenin signal transduction plays a tumor suppressive role in MB (36). Jiang et al. Found similar results. In human glioma, RBM5 inhibits Wnt/β- Catenin signal transduction to play a role in tumor inhibition (37). Rbm10 can inhibit Notch signal transduction and cell proliferation by regulating the variable splicing of numb. RBM10, a splicing factor, inhibits cell proliferation by switching hTERT transcripts to generate a function-less isoform and suppressing the telomerase activity in pancreatic cancer (38). RBM10 is also an alternative splicing regulator of the Notch regulator gene NUMB. Jordi Hernandez et al. found that RBM10 can inhibit cell proliferation by promoting exon 9 skipping of NUMB in lung adenocarcinoma (LUAD) (39). The inhibitory effect of RBM10 on cell proliferation can be obtained through inactivating RAP1/Akt/CREB signaling pathway in LUAD cells (40).

Other RBM proteins family members also participate in suppressing proliferation in different cancers, such as RBM6, RBMS1, RBMS2, RBMS3, etc. (**Table 2**). In fact, many RBM proteins family members have dual effects on tumor cells, namely promoting proliferation and inhibiting proliferation, including RBM3, RBM4, RBM10, RBMX, etc. The mechanism of these genes’ dual effect in different tumors is not precise. It may be related to the characteristics of tumors and the location of gene expression, still need more research.

**Table 2:** RBM family proteins effect as an inhibitor in cancers.

RBM family protein	Cancers	The role of RBMs in the cancer	Relative molecular mechanism	Associate cell lines or animal models	PMID
RBM5	Bladder cancer	RBM5 promotes the apoptosis of bladder cancer cells.	The down-regulation of RBM5 activates β-catenin, which binds to the T-cell factor/lymphocyte enhancer factor element of the miR-432-5p promoter and elevates the expression of miR-432-5p in bladder cancer cells.	Bladder cancer cell lines (T24, UM-UC-3, J82, and RT4)	31318608
	Gastric cancer	RBM5 inhibits gastric cancer cell proliferation	RBM5 decreased p53 transcriptional activity. And RBM5 silencing reduced the messenger RNA and protein expression of the p53 target gene p21.	Gastric cancer cell lines (MKN45)	28347247
	Gliomas	RBM5 inhibits cell proliferation, migration and invasion.	RBM5 plays a suppressor role in human gliomas by inhibiting Wnt/β-catenin signaling and inducing cell apoptosis.	Glioma cell lines (SHG44, U87, U251)	28061901
	Medulloblast-oma	RBM5 inhibited cell proliferation and migration of medulloblastoma.	RMB5 inhibits tumorigenesis of gliomas through inhibition of Wnt/β-catenin signaling.	Medulloblastoma cell lines (Daoy cells and ONS-76 cells)	32610314
	Lung cancer	RBM5 can inhibit the growth of lung cancer cells and induce apoptosis.	RBM5, by decreasing Bcl-2 expression, could induce caspase-3, caspase-9, PAPP cleavage and promoted apoptosis.	Lung cancer cell lines (A549)	22866867
	Lung cancer	RBM5 as a tumour suppressor in the mouse lung.	RBM5 acts in vivo as a tumor suppressor that likely underpins at least part of the pro-tumourigenic outcomes resulting from 3p21.3 deletion in humans.	The Rbm5 gene trap mouse line	29176597
	Lung cancer	RBM5 inhibited tumor growth.	Although RBM5's involvement in the death receptor-mediated apoptotic pathway is still to be investigated, RBM5-mediated growth suppression, at least in part, employs regulation of the mitochondrial apoptotic pathways.	Lung cancer cell lines (A549)	23721095
	Lung cancer	RBM5 expression loss may increase the metastatic potential of tumors.	RBM5 can regulated the genes involved in the functions of cell adhesion, migration and motility, known to be important in the metastatic process.	The parental cell lines (A549, Calu-6, NCI-H1299, BEAS-2B, and MCF-10A)	20338664
	Small cell lung cancer	RBM5 expression slows SCLC cell line growth, and increases sensitivity to the chemotherapy drug cisplatin.	RBM5 may play a direct role in regulating the cell cycle and apoptosis in SCLC cells.	Small cell lung cancer cell lines (GLC20)	27957556
	prostate cancer	RBM5 inhibited proliferationand induced apoptosis of PC-3 cells	RBM5 may induce the apoptosis of prostate cancer PC-3 cells by modulating the mitochondrial apoptotic pathway	Prostate cancer cell lines (PC-3)	23158838
RBM6	Laryngocarci-noma	RBM6 represses the growth and progression in laryngocarcinoma.	Upregulation of RBM6 reduced the expression of EGFR, ERK and p-ERK in vitro and in vivo.	Laryngocarcinoma cell lines (TU212, M4E, M2E and Hep-2) and normal nasopharyngeal epithelial cell line (NP69)	30772516
RBM38	Breast cancer	RBM38 overexpression counteracted cell migration and invasion induced by TGF-β in breast cancer cells	Transforming growth factor-β induced a remarkable downregulation of RBM38 in breast cancer that was directly regulated by transcription repressor Snail targeting the E-box elements in promoter region of RBM38 gene. Additionally, RBM38 positively regulated ZO-1 transcript via directly binding to AU/U-rich elements in its mRNA 3'-UTR.	Breast cancer cell lines (MCF7, BT474, and MDA-MB-231)	28683467
	Breast cancer	RBM38 acts as a tumor suppressor of breast cancer.	RBM38 destabilized the c-Myc transcript by directly targeting AU-rich elements (AREs) in the 3′-untranslated region (3′-UTR) of c-Myc mRNA to suppress c-Myc expression. Moreover, specific inhibitors of c-Myc transcriptional activity inhibited RBM38-induced suppression of growth, implying that RBM38 acts as a tumor suppressor via a mechanism that depends, at least partially, on the reduction of c-Myc expression in breast cancer.	Breast cancer cell lines (MCF-7 and ZR-75-1)	28399911
	Breast cancer	RBM38 could suppress breast cancer cells metastasis.	RBM38 promotes competing endogenous RNA (ceRNA) network crosstalk among STARD13, CDH5, HOXD10, and HOXD1 (STARD13-correlated ceRNA network), which we previously confirmed in breast cancer cells through stabilizing the transcripts and thus facilitating the expression of these four genes in breast cancer cells.	Breast cancer cell lines (MCF-7 and MDA-MB-231)	29733656
	Breast cancer	RBM38 could inhibit breast tumor cell proliferation, migration and invasion.	RBM38a up-regulate E-cadherin and down-regulate vimentin protein expression in breast cancer cells	Breast cancer cell lines (MCF-7, MDA-MB-231, BT474 and ZR-75) and non-malignant breast epithelial cells (MCF-10A)	24884756
	Breast cancer	RBM38 could suppress the growth of breast cancer cells.	PTEN was positively regulated by RBM38 via stabilizing its transcript stability, which in turn alleviated RBM38-mediated growth suppression.	Breast cancer cell line (BT474, MDA-MB-453)	29052531
	Colorectal cancer	RBM38 repressed colorectal cancer progression in vitro and in vivo	RBM38 inhibits colorectal cancer progression by competitively binding to PTEN 3'UTR with miR-92a-3p.	CRC cell lines (SW1116, SW480, HCT15, and SW620) and colonic epithelial cells (NCM-460)	34453780
	Endometrial Cancer	RBM38 overexpression attenuated the stemness of endometrial cancer spheres.	RBM38 overexpression activated the Hippo pathway through directly binding to MST1/2. Inhibition of MST1/2 rescued RBM38-mediated effects on endometrial cancer sphere stemness.	Endometrial cancer cell lines AN3CA, KLE, HEC-1A, and HEC-1B cells	32088727
	Hepatocellular carcinoma	RBM38 could inhibit cell migration and invasion.	HOTAIR could promote migration and invasion of HCC cells by inhibiting RBM38, which indicated critical roles of HOTAIR and RBM38 in HCC progression.	Liver cancer cell lines (HepG2 and Bel-7402)	24663081
	Hepatocellular carcinoma	Ectopic expression of RBM38 could induce liver cancer cell apoptosis and senescence, inhibit proliferation and colony growth, and suppress migration and invasion in vitro.	RBM38 may be a core contributor in stabilizing the p53-mdm2 loop function to prevent HCC, and a potential novel target to provide a therapeutic strategy for HCC by inhibiting mdm2 and rescuing p53 from inactivation.	Liver cancer cell lines (BEL-7402, BEL-7404, SMMC-7721, MHCC-97H, MHCC-97 L, HepG2, HCCLM3, and Hep-3B) and normal liver cells (L02)	30176896
	Lymphoma	Rbm38 functions as intergenic suppressors in aging and tumorige-nesis	Rbm38 and p63 form a feedback regulatory loop. In addition, mice deficient in Rbm38 or TAp63 are prone to spontaneous tumors. Rbm38 deficiency extends the lifespan and reduces tumor penetrance in TAp63+/− mice.	Rbm38-conditional knockout mice	29520104
	Lymphoma	Rbm38 significantly alters cancer susceptibility in mutant p53 knock-in mice by shortening lifespan, altering tumor incidence, and promoting T-cell lymphomagenesis.	Loss of Rbm38 enhanced mutant p53 expression and decreased expression of the tumor suppressor Pten, a key regulator of T-cell development. Rbm38 controls T-cell lymphomagenesis by jointly modulating mutant p53 and Pten.	Rbm38-knockout mice; tumor cell lines (U2OS, HCT116, and MiaPaCa2 cells)	29330147
	Non-small cell lung cancer	Overexpression of RBM38 inhibited non-small cell lung cancer cells proliferation, migration and invasion, and promoted cells apoptosis.	RBM38 could increase CASC2 expression via competitively binding to CASC2 with miR-181a. NPC1 inhibits NSCLC progression at least partly through miR-181a/CASC2 axis.	NSCLC cell line (A549)	29288351
	Osteosarcoma	Rbm38 attenuates E2F1-mediated cell-cycle progression.	E2F directly regulates expression of Rbm38 in a p53-independent manner. Binding of E2F1 to the RBM38 promoter was significantly enhanced upon ER-E2F1 activation.	Osteosarcoma cells (U2OS and SAOS-2)	22798430
	Renal cell carcinoma	RBM38 represses renal cancer cell proliferation, migration, and invasion	RBM38 inhibited RCC cell lines migration and invasion through EMT suppression, which may occur not only by up-regulating E-cadherin but also by down-regulating mesenchymal genes, such as β-catenin. 40 However, additional studies are required to fully understand the detailed function of RBM38 in EMT.	RCC cell lines (CAKI-1 and CAKI-2)	28459215
RBM43	Hepatocellular carcinoma	Overexpression of RBM43 suppressed cell proliferation in culture and resulted in the growth arrest of tumor xenografts.	RBM43 directly associated with the 3'UTR of Cyclin B1 mRNA and regulated its expression.	HCC cell lines (Hep3B 2.1-7, HepG2, QGY7703, and SMMC-7721); Rbm43−/− mice	32632220
RBMS1	Colon cancer	RBMS1 is a suppressor of colon cancer metastasis	RBMS1 as a post-transcriptional regulator of RNA stability with broad functional consequences for the transcriptome and clear implications for CRC progression.	CRC cell lines (SW480, LS174T, WiDr, HCT116, and COLO320)	32513775
	Prostate cancer	Overexpression of RBMS1 in prostate cancer cells resulted in diminished cell proliferation, colony forming ability as well as in retarded gap closing.	miRNA-106b interacts with the RBMS1 3′UTR and inhibits protein expression in PCa cell lines.	Prostate cancer cell lines (LNCaP and DU145)	33093529
RBMS2	Breast cancer	RBMS2 inhibited the proliferation of breast cancer	RBMS2 stabilized the mRNA of P21 by directly binding to the AU-rich element of 3'-UTR region. Anti-proliferation activity induced by overexpression of RBMS2 was rescued by interfering with the expression of P21.	Breast cancer cell lines (MCF-7, ZR-75-1, SK-BR-3, MDA-MB-453 MDA-MB-231 and MCF-10A)	30514345
RBMS3	Breast cancer	Ectopic expression of RBMS3 contributed to inhibition of cell migration, invasion in vitro and lung metastasis in vivo.	RBMS3 negatively regulated Twsit1 expression via directly binding to 3′-UTR of Twist1 mRNA, and thereby decreased Twist1-induced expression of matrix metalloproteinase 2 (MMP2).	Breast cancer cell lines (MDA-MB-231, MDA-MB-453, SUM-1315, SKBR3 and ZR-75-1)	30819235
	Breast cancer	RBMS3 suppresses the proliferation, migration, and invasion of breast cancer cells	RBMS3 greatly inhibited the protein expression of β-catenin, cyclin D1, and c-Myc in breast cancer cells.	Breast cancer cell lines (MCF-7, MDA-MB-231, and BT-474), and normal human breast epithelial cell line (NBEC)	28409548
	Nasopharyngeal carcinoma	RBMS3 has a strong tumor suppressive role in nasopharyngeal carcinoma.	RBMS3 was associated with its role in cell cycle arrest at the G1/S checkpoint by upregulating p53 and p21, downregulating cyclin E and CDK2, and the subsequent inhibition of Rb-ser780. Further analysis demonstrated that RBMS3 had a pro-apoptotic role in a mitochondrial-dependent manner via activation of caspase-9 and PARP. Finally, RBMS3 inhibited microvessel formation, which may be mediated by down-regulation of MMP2 and β-catenin and inactivation of its downstream targets, including cyclin-D1, c-Myc, MMP7, and MMP9.	NPC cell lines (C666, SUNE1, CNE2) and one immortalized nasopharyn-geal epithelial cell lines (NP460)	22957092
	Gastric cancer	RBMS3 inhibit cell proliferation and the GC cell cycle progression.	The microvessel density is closely related to RBMS3 and nuclear HIF1A expression in GC. down regulation of RBMS3, along with up regulation of nuclear HIF1A could act as a novel therapeutic molecular target for GC and might promote angiogenesis in GC.	GC cell lines (AGS, BGC-823 and MKN-45)	27902480
RBMS3-AS3	Prostate cancer	BMS3-AS3 suppresses cell proliferation, migration, invasion, and angiogenesis as well as the tumorigenic ability of prostate cancer.	RBMS3-AS3 acts as a miR-4534 sponge to inhibit the development of prostate cancer by upregulating VASH1.	prostate cancer cell lines (PC-3, DU145,LNCap, C4-2) and normal prostate cell line (PWPE-1)	31712637

## The Effect of RBM Proteins Family on Tumor Cell Apoptosis

The studies mentioned above indicate that the RBM proteins family members play essential roles in tumor proliferation. In addition, many studies have found that the RBM proteins family also involve in the regulation of apoptosis in cancer, mainly in two aspects.

### RBM Proteins Family Can Regulate Tumor Cell Apoptosis by Induced Pro-Apoptotic Genes and Apoptosis Regulatory Proteins, Including Bax and p53

Garabito et al. found that the expression of RBM genes (RBMX, RBM3, and RBM10) on the X chromosome is remarkably associated with the pro-apoptotic gene Bax in breast cancer cells (41). RBM10, a vital member of the RBM genes on the X chromosome, can also promote cell apoptosis by enhancing the expression of TNF-α and regulating the alternative splicing of related genes, including FAS and BCL-X (42, 43). Rbm10 can also increase the stability of p53 by inhibiting MDM2 mediated ubiquitination and degradation of p53 and prolong the half-life of p53 to induce apoptosis to inhibit cancer cell proliferation and induce apoptosis.

### RBM Proteins Family Can Affect Apoptosis by Enhanced Mitochondrial Apoptotic Activity and Upregulated the Expression of Autophagy-Related Proteins

Zhao et al. found that RBM5 protein expression significantly decreased in prostate cancer tissues than in normal tissues. Mitochondrial apoptotic activity is significantly increased when RBM5 is overexpressed in prostate cancer cells (44). The upregulation of RBM5 can induce cell apoptosis and increased cell sensitivity to certain apoptotic stimuli by altering the apoptosis regulatory proteins (44, 45). Loiselle et al. reported that RBM5 could directly regulate the cell cycle and apoptosis in small-cell lung cancer (SCLC) (46). RBM5 upregulates the level of autophagy-related proteins, such as LC3, Beclin1, and LAMP1, which further induce cell autophagy in LUAD (47). Similarly, down-regulation of RBM5 in bladder cancer cells leads to inhibition of apoptosis by increasing the expression of β-catenin-mediated mir-432-5p (48). The pro-apoptotic effect of RBM protein may contribute to inhibit tumor progression. Hence, it can accelerate tumor cell death by inducing the expression of RBM proteins family members. And this mechanism may benefit targeted therapy of tumors in the future.

## The RBM Proteins Family Affect the Invasion and Migration of Tumor Cells

In addition to playing a role in tumor cell proliferation and apoptosis, the RBM proteins family can also affect the migration and invasion of tumor cells. Most RBM proteins family effect as an inhibitor in the invasion and metastasis of cancer, the primary biology mechanism as the following.

### RBM Proteins Family Protein Can Inhibit Tumor Cell Invasion and Metastasis by Targeted Gene Expression

Zonula occludens-1 (ZO-1) is a member of the membrane-associated guanylate kinase (MAGUK) family of proteins, which can control endothelial cell-cell tension, cell migration, and barrier formation (49). RBM38 can positively regulate the ZO-1 gene by directly binding to AU/U-rich elements in the ZO-1 mRNA 3’-UTR. Therefore, overexpression of RBM38 can reverse the invasion and migration of breast cancer cells caused by the knockdown of ZO-1 (50). RBMS3 negatively regulates the expression of Twsit1 and reduces the level of Matrix metalloproteinase 2 (MMP2) induced by Twist1, thus inhibiting the invasion and metastasis of breast cancer cells (51). In human prostate cancer, RBM25 binding directly to an Amotl1-derived circRNA, circAMOTL1L, resulted in the relief of the miR-193a-5p repression of the Pcdha gene cluster, whereas p53 regulates EMT *via* directly activating the RBM25 gene (52).

### RBM Proteins Family Could Inhibit Cancer Invasion and Metastasis by Involving Signaling Pathways, and Regulation mRNA Stability, etc

RBM47 could inhibit the metastasis of NSCLC by increasing the stability of AXIN1 mRNA and then inhibiting Wnt/β-catenin signal transduction (53). In breast cancer, RBM47 also plays a similar role. Dickkopf-1(DKK-1), as a WNT signaling pathway inhibitor 1, was bound with RBM47 to inhibit the activation of the WNT pathway and exert a tumor suppressor effect (54). As a post-transcriptional regulator of RNA stability, RBMS1 has clear significance for the progression of colon cancer. In a mouse model of xenotransplantation, silencing RBMS1 increased the metastatic ability of colon cancer cells while restoring RBMS1 weakened the metastatic capacity of colon cancer cells (10). Some studies have also shown that RBM5 could inhibit the metastasis and invasion of lung cancer (55, 56). Moreover, RBM3 downregulation is related to the distant metastasis of esophageal squamous cell carcinoma (57).

### RBM Proteins Family Also Can Promote Cancer Cell Invasion and Metastasis

However, Huang et al. reported that RBM4 promotes the migration and invasion of esophageal cancer. They found that RBM family protein could promote tumor invasion and metastasis by participating in the alternative splicing of some genes, such as tropomyosin I (TPM1). Knockout of the RBM4 gene resulted in specific down-regulation of TPM1 variants V2 and V7, which might inhibit migration and filamentous group formation in esophageal cancer cells (58). RBM5-AS1 can be used as an oncogenic factor in multiple cancers, such as hepatocellular carcinoma, osteosarcoma, and oral squamous cell carcinoma. Mu et al. showed that RBM5-AS1 could decrease miR-132/212 by recruit PRC2 complex, and facilitate HCC cell migration and invasion (59). Fu et al. found that RBM11 was highly expressed in ovarian cancer and could promote tumor cell invasion and metastasis by activating Akt/mTOR signaling (60). circRBM33 was generated from the RBM33 and could promote gastric cancer cells migration and invasion through the circRBM33/miR-149/IL-6 axis (61).

In conclusion, RBM proteins family protein has a dual role in different cancers. Most RBM proteins family effect as an inhibitor in the migration and invasion of cancer cells, while a small group of RBM proteins family members could facilitate tumor cell migration and invasion. It is necessary to explore further why RBM proteins family play different roles in tumor cells. And the relative molecular mechanism of the RBM proteins family may provide a theoretical basis for future research and clinical application.

## RBM Proteins Family Can Use As a Predictor for a Prognosis of Cancer

Recently, it was reported that in various tumors, such as liver cancer, gastric cancer, lung cancer, and breast cancer, the expression level of RBM proteins is related to the tumor size, invasion depth, lymph node metastasis, and prognosis. Yong et al. found that RBM4 expression in gastric cancer tissues was significantly lower than that in adjacent normal tissues. The downregulation of RBM4 was significantly associated with poor differentiation, lymph node metastasis, distant metastasis, and advanced Tumor Node Metastasis (TNM) stage in gastric cancer (62). They found that compared with the RBM4 high-expression group, the RBM4 low expression group had worse overall survival (OS) and disease-free survival (DFS) (62). Gao et al. showed that the overexpression of RBM3 in patients with colorectal cancer, gastric cancer, or melanoma predicted a good prognosis (63). Besides, RBM15 was identified as high-confidence interactors with Wilms tumor-associated protein (WTAP) in proteomic analysis. WTAP binds METTL3, the methyltransferase that mediates methylation of m6A in mRNA16, and is recruited to RNAs *via* an unknown adaptor protein to trigger m6A formation. Patil et al. found RBM15 is part of the WTAP-METTL3 N6-methyladenosine (m6A) methyltransferase complex and participates in M6A modification (64). Studies on RNA methylation regulators in papillary thyroid carcinoma (PTC) and gastric cancer have shown that RBM15 was significantly positively correlated with a better prognosis (65, 66). But in LUAD, the high expression level of RBM15 is related to a poor prognosis (67, 68).

**Figure 2:**
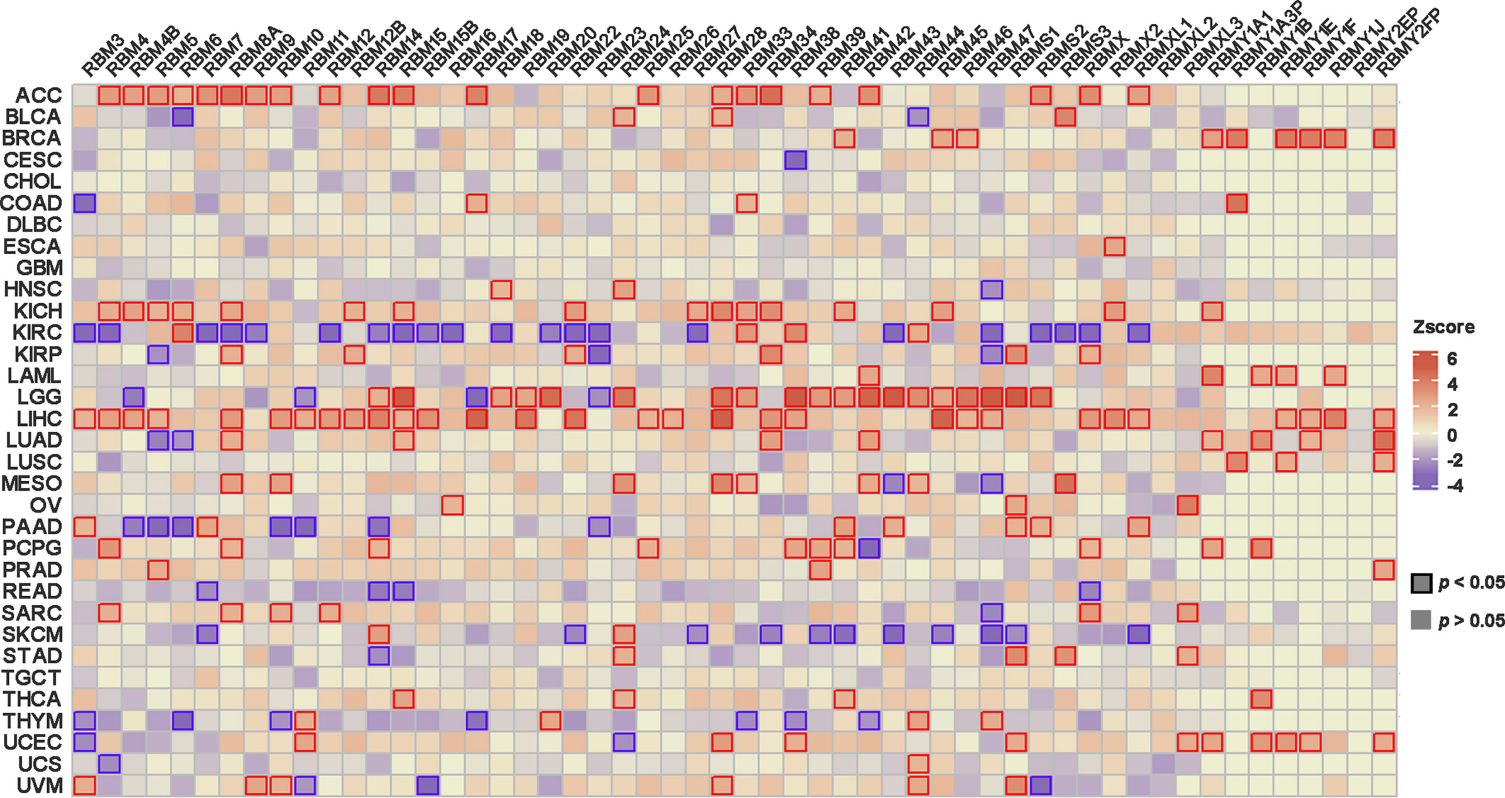
The correlation analysis between RBM proteins family expression and the patient’s prognosis in the TCGA. *p < *0.05 was considered statistically significant.

And we further explored the relationship between the expression level of RBM proteins family members and prognosis in different cancers by TCGA. The results showed that members of the RBM proteins family were significantly correlated with tumor prognosis, and the expression levels of many RBM members could predict the prognosis of tumor patients (**Figure 2**). For example, a high expression of the RBM proteins was associated with shorter survival in ACC. Conversely, low expression of the RBM proteins in kidney renal clear cell carcinoma (KIRC) was associated with a worse prognosis.

## RBM Can Be Used as a Potential Therapeutic Target in Cancers

As mentioned above, there are significant differences in the expression levels of RBM proteins in lung cancer, breast cancer, liver cancer, colon cancer, and other human cancers, and their expression levels are significantly correlated with prognosis. Therefore, targeting RBM protein may be a new therapeutic strategy for the treatment of human cancer. In fact, previous studies have shown that RBMX is highly expressed in HCC tissues and cell lines, resulting in increased drug resistance of HCC cells (69, 70). And targeting RBMX can be used as a new strategy for HCC treatment. RBM39 can bind to c-Jun and stimulate its transcriptional activity, promoting its involvement in many aspects of cancer development. Studies have found that RBM39 is highly expressed in breast cancer tissues and can promote tumor cell proliferation. Considering the role of RBM39 in breast cancer, Shannon D Chilewski et al. developed an RBM39 peptide to treat triple-negative breast cancer (71). A bioinformatics analysis also showed that RBM39, a target gene of miR-494, can be used as a biomarker to predict trastuzumab resistance in breast cancer (72). Additionally, Wu et al. found that loss of RBMS3 might increase the chemical resistance of epithelial ovarian cancer (73). Downregulation of mir-383 induced RBM24 mediated NF-κB signal activation. Therefore, RBM24 can become a potential therapeutic target to reverse the chemoresistance of lung adenocarcinoma cells (74). In patients treated with oxaliplatin, a first-line chemotherapy drug, high expression of RBM3 is an independent predictor of prolonged survival in patients with metastatic colorectal cancer (75). In epithelial ovarian cancer cell lines, RBM3 expression silencing resulted in decreased sensitivity to cisplatin. It was suggested that RBM3 might be a useful therapeutic predictor in epithelial ovarian cancer (76).

Although the role of other members of the RBM proteins family in tumor treatment and prognosis is not clear, existing studies have shown that some members of RBM proteins family, such as RBM3, RBM4, and RBM39, play an essential role in tumor treatment and prognostic markers. Therefore, RBM proteins may be a potential target for tumor treatment and prognosis.

## Conclusion

In recent years, increasing attention has been paid to the RBM proteins family, and their various roles in multiple cancers have been continuously revealed. It was shown that some members of the RBM proteins family play a tumor-suppressive role in cancers, inhibiting tumorigenesis and cell proliferation, promoting tumor cell apoptosis, and limiting cell migration and invasion, such as RBM6 and RBM38. While some members play the opposite role, promoting cell proliferation and the invasion of cancer, including RBM7, RBM11, and RBM15. Besides, another part of RBM proteins plays a dual function of cancers (**Table 3**). For instance, RBM3 plays a cancer-promoting role in breast and colorectal cancer, while it inhibits tumorigenesis in prostate cancer. And RBM5 and RBM5-AS1 play opposite effects in tumor cells. RBM5 can inhibit tumor cell growth in gastric cancer and lung cancer. RBM5-AS1 promotes the migration and invasion of osteosarcoma tumor cells. It was not clarified that why RBM proteins family members play a dual role in tumors. Kido et al. found that the dual role of RBM proteins family genes may be related to time and space (77). They discovered that RBMY acts as a suppressor in the early stages of the tumor and shows a cancer-promoting effect in the long-term progression of tumors. However, the specific mechanism still needs more in-depth exploration. The dual function of RBMs may provide a novel idea for the treatment and research of tumors, as some tumor-promoting factors may also be turned into tumor suppressor factors under some conditions. Besides, it can also amplify the tumor suppressor role of RBM protein that may be used as a new target for clinical treatment of tumors. In future studies, further exploration of the dual role of RBM proteins family in tumors and elucidating the related molecular mechanisms may contribute to the development of new therapeutic targets.

**Table 3:** RBM family proteins serves dual functions in cancers.

RBM family protein	Cancers	The role of RBMs in the cancer	Relative mechanism	Associate tumor cell lines or animal models	PMID
RBM3	Breast cancer	RBM3 promotes the proliferation and metastasis of human breast cancer cells.	RBM3 regulates ARPC2 in a post-transcriptional 3′UTR-binding manner to promote breast cancer cells proliferation and migration.	Human normal breast cells (MCF10A and HMEC);	30720048
				breast cancer cells (MCF7, T47D, MDA-MB-468, BT474, MDA-MB-231 and BT549)	
	Cancers	RBM3 promotes directional cell migration.	The RBM3 expression induce changes in cell polarity and spreads, involving RhoA-Rho associated protein kinase (ROCK) signaling and the collapsin response mediator protein 2 (CRMP2).	B104 neuroblastoma, NIH 3T3, N2A and HeLa cell lines	29743635
	Colorectal cancer	RBM3 is the central regulator of tumorigenesis, depletion of which enhances the regression of tumors.	RBM3 knockdown increases caspase-mediated apoptosis coupled with nuclear cyclin B1, and phosphorylated Cdc25c, Chk1 and Chk2 kinases. And RBM3 enhances COX-2, IL-8 and VEGF mRNA stability and translation.	CRC cell lines (HCT116, SW480), cervical carcinoma cells (HeLa) and mouse fibroblast cells (NIH3T3)	18427544
	Colorectal cancer	RBM3 overexpression enhances stemness in cancer cells.	Upon RBM3 overexpression, β-catenin transcriptional activity is increased resulting in higher DCLK1+ and LGR5+ stem cell population thereby enhancing side population and spheroid formation.	CRC cell lines (HCT 116 and DLD-1)	26331352
	Hepatocellular carcinoma	RBM3 promotes the prolife-ration of HCC cells	RBM3 could promote YAP1 expression in HCC cells.	HCC cell lines (Huh7, SK-Hep1 and BEL-7404, Hep3B)	32976820
	Hepatocellular carcinoma	RBM3 promotes HCC cell proliferation.	RBM3 promoted the HCC cell proliferation in a SCD-circRNA 2 dependent manner.	HCC cell lines (Huh7, HepG2, HCT-15 and NCI-N87)	31235426
	Pancreatic cancer	RBM3 enhanced cell migration and invasion of pancreatic cancer.	Silencing of RBM3 did not influence levels of neither COX-2 nor IL-8, and there was no correlation between baseline levels of RBM3 and COX-2 or IL-8. Further studies are required to find other candidate targets and downstream effects of RBM3.	PrCa cell lines (BxPC-3, PANC-1 and MIAPaCa-2) and human fetal foreskin fibroblasts (HFFF2)	29464046
	Prostate cancer	RBM3 attenuated prostate cancer stem cell-like properties and tumorigenic potential.	RBM3 contributed to stem cell-like character in prostate cancer by inhibiting CD44v8-v10 splicing	Prostate cancer cell lines (PC3 and DU145)	23667174
RBM4	Breast cancer	SRPK1-RBM4 network modulated the sensitivity of breast cancer cells toward pro-apoptotic agents.	Breast cancer cells are deprived of apoptotic resistance through the RBM4-mediated up-regulation of the IR-B and MCL-1S transcripts.	Breast cancer cell line (HBL100 cells and MCF-7)	25140042
	Esophageal cancer	The capacity for cell migration was inhibited after RBM4 knockdown.	A natural antisense TPM1-AS regulates the alternative splicing of TPM1 through an interaction with RBM4 and involves in TPM1-mediated filopodium formation and migration of cancer cells.	Esophageal squamous cell line (SHEEC, KYSE140, KYSE150, KYSE180, KYSE450 and KYSE510)	28754317
	Cancers	RBM4 inhibits cancer cell proliferation and migration.	RBM4 regulates Bcl-x splicing to induce apoptosis, and coexpression of Bcl-xL partially reverses the RBM4-mediated tumor suppression. Moreover, RBM4 antagonizes an oncogenic splicing factor, SRSF1, to inhibit mTOR activation.	Lung cancer (H157), breast cancer (MDA-MB-231), ovarian cancer (SKOV3), panceatic cancer (Panc-1), liver cancer (HepG2), and prostate cancer (PC-3)	25203323
	Gastric cancer	RBM4 inhibits gastric carcinoma cell cell proliferation, migration and invasion.	RBM4 was involved in the activation of MAPK-dependent signaling pathways in human GC.	Normal gastric cell line (GES1) and human gastric carcinoma cell lines (MKN28, HGC27, BGC823, MKN45, and MGC803)	31145716
	Non-small cell lung cancer	RBM4 inhibits NSCLC cells prolife-ration ability.	RBM4 was responsible for NSCLC progression regulated by USP3.	NSCLC cell lines (H1299 and SPCA1)	32271432
RBM10	Lung cancer	RBM10 promoted cell growth and proliferation and increased cell migration.	RBM10 activated key proliferative signaling pathways [such as the epidermal growth factor receptor (EGFR), mitogen-activated protein kinase (MAPK) and phosphoinositide 3-kinase (PI3K)-AKT pathways] and inhibited apoptotic pathways.	Lung adenocarcinoma cell lines (A549 and H1299) and human lung fibroblast cells (HLF)	30483773
	Colon cancer	Overexpression of RBM10 inhibits cancer cell proliferation, migration and mitochondrial respiration and promotes apoptosis.	RBM10 induces apoptosis partly by inducing p53 and activating its activity. And RBM10 can increase p53 stability by inhibiting MDM2-mediated p53 ubiquitination and degradation.	CRC cell lines (HCT116, H460), U87 and MCF7	31591476
	Hepatocellular carcinoma	RBM10 attenuates proliferative and invasive abilities, but drives apoptosis in HCC cells, thus alleviating the progression of HCC.	Overexpression of RBM10 downregulated protein levels of EGFR and p-ERK in HCC-LM3 and HepG2 cells.	HCC cell lines (HepG2 and HCC-LM3)	32572914
	Lung cancer	RBM10 inhibits tumor cell growth of mouse tumor xenografts	RBM10 represses Notch signaling and cell proliferation through the regulation of NUMB alternative splicing.	NSCLC cell lines (A549)	26853560
	Lung cancer	RBM10 overexpression suppresses lung cancer cell proliferation.	RBM10 decreases the activation of RAP1 and reduces the phosphorylation of CREB via the AKT signalling pathway, suggesting that RBM10 exhibits its effect on lung adenocarcinoma cell proliferation via the RAP1/AKT/CREB signalling pathway.	NSCLC cell lines (A549 and H1299)	30955253
	Lung cancer	RBM10 overexpression inhibited viability and colony formation of lung adenocarcinoma cancer cells	RBM10 regulates many gene pathways involving in the tumor development or progression, such as focal adhesion, peroxisome proliferator-activated receptor-regulated gene pathway, cytokine-cytokine receptor interaction, mitogen-activated protein kinase signaling, complement and coagulation cascades.	Lung adenocarcinoma cell lines (A549 and H1299)	28347232
	LUAD	RBM10 can suppress LUAD development and progression.	RBM10 mutation-associated AS events identified in LUADs are largely induced by RBM10 loss. RBM10-mediated regulation of EIF4H exon 5 splicing led to consistent changes at protein levels and RNA levels. RBM10-mediated splicing switch of EIF4H plays a critical role in regulating LUAD progression.	Lung adenocarcinoma cell lines (A549, PC9, H1975 and H1944)	33130397
	Osteosarcoma	RBM10 decreased the tumor cell proliferation, colony formation, migration and invasion.	RBM10 overexpression induced osteosarcoma cell apoptosis via the inhibition of Bcl-2, the activation of caspase-3, and the transcription and production of TNF-α.	Osteosarcoma cell lines (U2OS)	30403180
	Pancreatic cancer	RBM10 inhibits cell prolife-ration, invasion, colony formation, and xenograft growth.	RBM10 promotes the exclusion of exons7 and 8 which results in the production of TERT-s transcripts.	PrCa cell lines (AsPX-1, BxPC-1, HPAC, PANC-1, CFPAC-1) and pancreatic ductal epithelial cell (HPDE)	33520366
RBM24	Bladder cancer	RBM24 promotes the proliferation of bladder cancer cells in vitro.	RBM24-regulated BC cell proliferation was moderated via the Runx1t1/TCF4/miR-625-5p feedback loop.	The normal uroepithelial cell (SV-HUC-1) and bladder cancer cell lines (UM-UC-3, 253 J, T24, and J82)	34021255
	Liver cancer	RBM24 inhibits liver cancer cell growth and progression and induces sorafenib sensitivity.	RBM24 inhibits nuclear translocation of CTNNB1 in liver cancer cells.	HCC cell lines (Huh7, Hep3B and HepG2)	34345299
	Nasopharyngeal carcinoma	RBM24 expression suppressed NPC cells proliferation, migration and invasion.	RBM24 inhibits the expression of MALAT1 through upregulation of the expression of miR-25, which directly targets MALAT1 for degradation.	Human NPC cell lines (NPEC1 cells)	27584791
RBM47	Nasopharyngeal carcinoma	RBM47 plays an oncogenic role in nasopharyngeal carcinoma cells	BM47 binds to the promoter and regulates the transcription of BCAT1, and its overexpression partially rescues the inhibitory effects of RBM47-knockdown on NPC cells. RBM47 promotes the progression of NPC through multiple pathways, acting as a transcriptional factor and a modulator of alternative splicing in cooperation with hnRNPM.	NPC cell lines (S26, 5-8F and HONE1)	34274258
	Breast cancer	RBM47 as a suppressor of breast cancer progression and metastasis.	RBM47 altered splicing and abundance of a subset of its target mRNAs. Some of the mRNAs stabilized by RBM47, as exemplified by dickkopf WNT signaling pathway inhibitor 1, inhibit tumor progression downstream of RBM47	Breast cancer cell lines (SKBR3, ZR-75-30 and HCC1954)	24898756
	Colorectal cancer	RBM47 inhibited CRC cell migration, invasion, and metastasis.	Activation of conditional SNAIL and SLUG alleles suppressed expression of RBM47 at the mRNA and protein levels in DLD1 CRC cells. RBM47 is also repressed by EMT-TFs, which are up-regulated in cancer cells during tumor progression and mediate EMT, thereby promoting invasion and presumably metastasis.	CRC cell lines (SW480, SW620, Caco-2)	28680090
	Lung cancer	RBM47 could inhibit the tumor formation and metastasis of xenografted mice.	RBM47 suppresses Nrf2 activity by upregulating KEAP1 and CUL3, and suppressing some Nrf2 activators (Figure 6), leading to the inhibition of tumor growth in vivo.	Human lung cance cell lines (A549, NCI-H441)	26923328
RBMX	Non-small cell lung cancer	RBM47 is capable of suppressing NSCLC metastasis and progression in vitro and in vivo.	BM47 could control the AXIN1/WNT/β-catentin signaling axis through stabilizing AXIN1 mRNA levels via direct 3′-UTR binding	NSCLC cell lines (H1299 and A549)	32891348
	Hepatocellular carcinoma	RBMX is significantly contributes to the tumorigenesis and sorafenib resistance of hepatocellular carcinoma.	RBMX through BLACAT1 induces tumorigenesis. The autophagy level and cancer cell stemness were also improved when RBMX/BLACAT1 upregulated.	The human normal hepatic cell (LO2) and HCC cell lines (HepG2, Huh-7, SMMC7721, Hep3B, HCCLM3 and MHCC97H)	33294259
	Myeloid leukemia	RBMX and RBMXL1 could contribute to leukemia develop_x005fment and maintenance.	RBMX/RBMXL1 loss alters chromatin compaction and structure in AML cells. And RBMX and RBMXL1 maintain the chromatin state that is essential for the survival of AML cells through their transcriptional regulation of CBX5.	Myeloid leukemia cell lines (MOLM13, KCL-22, Kasumi-1, K562, THP-1, U-937, TF-1, NB4, HL-60, KG-1, Nomo-1); Rbmx^f^ sperm mice	34458856
RBMY	Bladder cancer	RBMX inhibited BCa cell proliferation, colony formation, migration, and invasion in vitro and suppressed tumor growth and metastasis in vivo.	RBMX competitively inhibited the combination of the RGG motif in hnRNP A1 and the sequences flanking PKM exon 9, leading to the formation of lower PKM2 and higher PKM1 levels, which attenuated the tumorigenicity and progression of BCa.	BCa cell lines (T24 and 5637)	33564070
	Hepatocellular carcinoma	Overexpression of RBMY impaired cell proliferation in the short term, while persistent RBMY expression promoted evolutionary adaptation and restoration of proliferative properties of the tumor cells.	RBMY may regulated genes that involved in various cell proliferative pathways, such as the RAS/RAF/MAP and PIP3/AKT signaling pathways.Y‐linked RBMY could serve dual tumor‐suppressing and tumor‐promoting functions, depending on the spatiotemporal and magnitude of its expression during oncogenic processes, thereby contributing to sexual dimorphisms in liver cancer.	HCC cell lines (HuH‐7 and HepG2 cells)	32473614
	Hepatocellular carcinoma	RBMY is a novel oncofetal protein, expresses generally in the developing and carcinogenic livers.	The Wnt–RBMY–GSK3b regulatory circuit represents a critical mechanism for oncogenic activation of b-catenin and malignant hepatic stemness.	HCC cell lines (HuH‐7 and HepG2 cells)	26185016
	Hepatocellular carcinoma	RBMY knockdown elicits inhibitory effects on the transformation and anti-apoptotic abilities of the human hepatoma cell.	The oncogenic mechanism of RBMY may be linked to its regulation of AR trans-activation activity by the increase of AR45 variant, the inhibitor of AR.	HCC cell lines (HepG2 and Hep3B)	22073224

In summary, based on the current research, the influence of the RBM proteins family on cancer is diverse, and these proteins are involved in various aspects of tumorigenesis and development. And RBM protein can be used as novel tumor markers for clinical application in early diagnosis and prognosis evaluation of multiple cancers.

## Author Contributions

ZL, QG and ZF searched the pubmed and literatures. JZ and QG performed tables and figures. QG, JZ, and ZF wrote the first draft of the manuscript. YW and ZL wrote sections of the manuscript. JT and TW review and editing manuscript. All authors contributed to the article and approved the submitted version.

## Conflict of Interest

The authors declare that the research was conducted in the absence of any commercial or financial relationships that could be construed as a potential conflict of interest.

## Publisher’s Note

All claims expressed in this article are solely those of the authors and do not necessarily represent those of their affiliated organizations, or those of the publisher, the editors and the reviewers. Any product that may be evaluated in this article, or claim that may be made by its manufacturer, is not guaranteed or endorsed by the publisher.

## Supplementary Material

Supplementary Table 1 The member of the RBM protein family.
